# A new species of the deuterostome *Herpetogaster* from the early Cambrian Chengjiang biota of South China

**DOI:** 10.1007/s00114-020-01695-w

**Published:** 2020-08-28

**Authors:** Xianfeng Yang, Julien Kimmig, Bruce S. Lieberman, Shanchi Peng

**Affiliations:** 1 Yunnan Key Laboratory for Palaeobiology, MEC International Joint Laboratory for Palaeobiology and Palaeoenvironment, Yunnan University, Kunming, 650091 China; 2 State Key Laboratory of Palaeobiology and Stratigraphy, Chinese Academy of Sciences, Nanjing, 210008 China; 3 Biodiversity Institute, University of Kansas, Lawrence, KS 66045 USA; 4 Earth and Mineral Sciences Museum & Art Gallery, Pennsylvania State University, University Park, PA 16802 USA; 5 Department of Ecology & Evolutionary Biology, University of Kansas, Lawrence, KS 66045 USA; 6 Nanjing Institute of Geology and Palaeontology, Chinese Academy of Sciences, Nanjing, 210008 China

**Keywords:** Burgess Shale-type preservation, Deuterostomes, Cambrian, Chengjiang biota, Chiungchussu Formation, Haiyan, Stalked filter feeder

## Abstract

The Cambrian radiation represents a key time period in the history of life. Here, we add to the mounting evidence accumulating on the nature of deuterostomes from this time period through description of a new species of stalked deuterostome, *Herpetogaster haiyanensis* nov. sp., from the lower Cambrian (series 2, stage 3) Chengjiang biota of China. This represents the first occurrence of the genus in Gondwana, the first juvenile specimen, and the oldest specimens to date. *Herpetogaster haiyanensis* nov. sp. differs from *H. collinsi* Caron et al. (2010) in having a stolon that is separated into an outer and inner layer, the segmentation of the body and in the shape and number of branches of the tentacles. The new species reiterates earlier suggestions of deuterostome affinities of the genus―it appears closely related to *Phlogites* and then successively more distantly related to *Cotyledon* and *Eldonia*―and may have fed on hyolithids.

## Introduction

The lower Cambrian (series 2, stage 3) Chengjiang biota of China is an exceptionally diverse Burgess Shale-type (BST) deposit that has been critical for illuminating the origin and evolution of many animal phyla, especially arthropods (Caron et al. 2014; Hou et al. 2017; Kimmig 2019). While arthropods, both soft-bodied and biomineralized, dominate the deposit, members of many modern phyla have also been reported; further, in many instances, these represent the first appearances of these phyla in the fossil record (Hou et al. 2017). The composition of the Chengjiang biota is similar to the middle Cambrian (Miaolingian; Wuliuan) Burgess Shale Lagerstätte of Canada, and previous research has shown that there are ecological and taphonomic similarities between the two deposits (Conway Morris 1986; Gabbott et al. 2004; Dornbos and Chen 2008; Caron et al. 2013, 2014; Zhao et al. 2014; Saleh et al. 2020). While few, if any species, are shared between the deposits, as they are from distinct biogeographic regions and temporally differentiated, several genera are shared between the Chengjiang biota and the Burgess Shale, as well as other Laurentian BST deposits, including *Anomalocaris*, *Cambroraster*, *Canadaspis*, *Choia*, *Eldonia*, *Isoxys*, *Leanchoilia*, *Sidneyia*, *Tuzoia*, and various trilobites (see Hendricks and Lieberman 2007; Vannier et al. 2007; Williams et al. 2007; Hendricks et al. 2008; Garcia-Bellído and Aceñolaza 2011; Caron et al. 2013, 2014; Hendricks 2013; Kimmig and Pratt 2015; Paterson et al. 2015a, b; Hou et al. 2017; Lerosey-Aubril et al. 2018, 2020; Harper et al. 2019; Kimmig et al. 2019a; Pates et al. 2019; and Liu et al. 2020 for a discussion of Cambrian BST deposits). Here we add to the list of genera shared between the Chengjiang and Laurentian BST deposits by reporting on a new species of the stalked deuterostome *Herpetogaster*, *Herpetogaster haiyanensis* nov. sp. It is known from at least eight individuals and was recovered from a new locality of the Chengjiang biota. *Herpetogaster collinsi* was first described from the Burgess Shale (Caron et al. 2010) and has recently also been identified in the lower Cambrian (series 2, stage 4) Pioche Formation of Nevada (Kimmig et al. 2019b). The new Chengjiang specimens extend the geographic range of the genus to Gondwana and also the record of soft-bodied deuterostomes in the Chengjiang biota.

## Geological setting

**Fig. 1:**
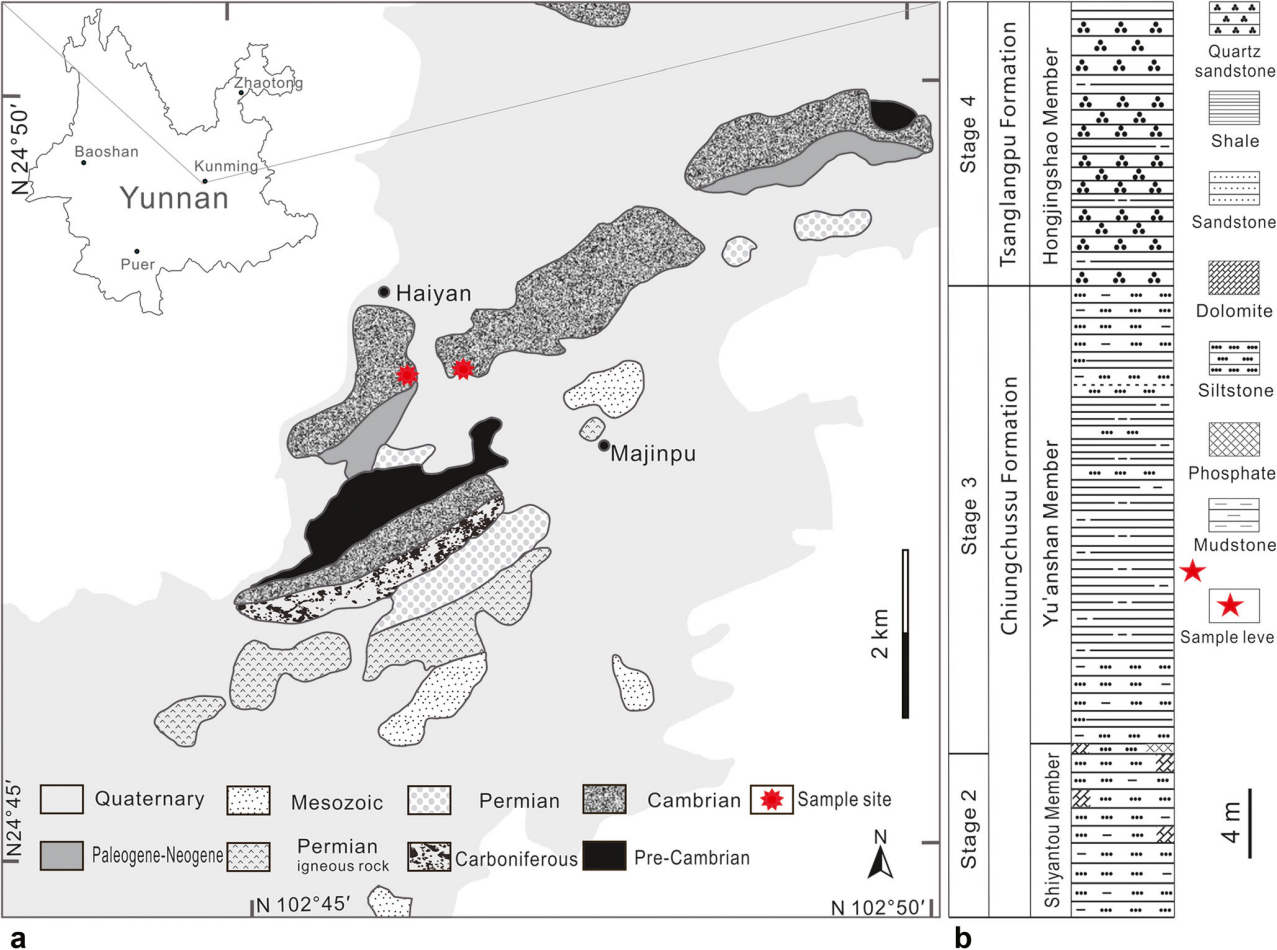
**a** Location of the Chiungchussu Formation near Haiyan. **b** Stratigraphy of the Chiungchussu Formation near Haiyan

The 35 cm^2^ slab, preserving the fossils described herein, is collected from the lowermost part of a new site hosting abundant soft-bodied fossils of the Chengjiang biota, herein referred to as the Haiyan section (Fig. 1a). The index trilobite, *Eoredlichia intermedia*, correlates to the lower Cambrian (series 2, stage 3) Yu’anshan Member of the Chiungchussu Formation (National Commission on Stratigraphy of China 2018). At the Haiyan locality, the Yu’anshan Member is ~ 25 m and is composed of finely laminated yellow mudstone intervals, interbedded with siltstones and sandstones (Fig. 1b). It unconformably overlies the siltstones of the lower Cambrian Shiyantou Member, Chiungchussu Formation, and is conformably overlain by the quartz sandstones of the lower Cambrian Hongjingshao Member of the Tsanglangpu Formation. To date 2846 specimens have been collected from the Haiyan locality, comprising at least 126 taxa belonging to 12 phyla.

## Materials and methods

The specimens were photographed using a Canon EOS 5D digital SLR camera equipped with a Canon 50 mm macro lens and cross-polarized lights. Close-ups were captured by using a Leica DFC 500 digital camera mounted on a Leica M205-C stereoscope. The contrast, colour, and brightness were adjusted using Adobe Photoshop CC. Line drawings were created in CorelDRAWX8. Specimens are housed in the collections of the Yunnan Key Laboratory for Palaeobiology, Yunnan University, Kunming, China (YKLP).

## Systematic palaeontology

Deuterostomia Grobben 1908

Ambulacraria Metschnikoff 1881

Cambroernida Caron, Conway Morris and Shu, 2010

*Herpetogaster* Caron, Conway Morris and Shu, 2010

*Type species*. *Herpetogaster collinsi* Caron, Conway Morris and Shu, 2010

*Species included*. *Herpetogaster collinsi* Caron, Conway Morris and Shu, 2010; *Herpetogaster haiyanensis* n. sp.

### Diagnosis

Segmented body, coiled dextrally. Short head bearing prominent bilateral anterior dendritic tentacles of sub-equal length and in two-by-two arrangement with pharyngeal structures, possibly lateral pores. Trunk sub-cylindrical, divided into two sub-sections, narrowing posteriorly. Ventral and contractile adhesive stolon, with terminal disc. Digestive tract with anterior mouth, pharynx, large stomach relative to the rest of the digestive tract, and narrow intestine with terminal anus. Stomach and intestine of sub-equal lengths, un-looped, with triangular mesenterial insertions. Based on Caron et al. (2010).

### Occurrence

Chiungchussu Formation, Yu’anshan Member, lower Cambrian (series 2, stage 3), *Eoredlichia*-*Wutingaspis* biozone, Haiyan section near Kunming, Yunnan Province, South China. Pioche Formation, Comet Shale Member, lower Cambrian (series 2, stage 4), *Nephrolenellus multinodus* biozone, Ruin Wash, Lincoln County, Nevada. Burgess Shale and Stephen Shale Formations, middle Cambrian (Miaolingian, Wuliuan), Yoho and Kootenay National Parks, British Columbia, Canada.

**Fig. 2:**
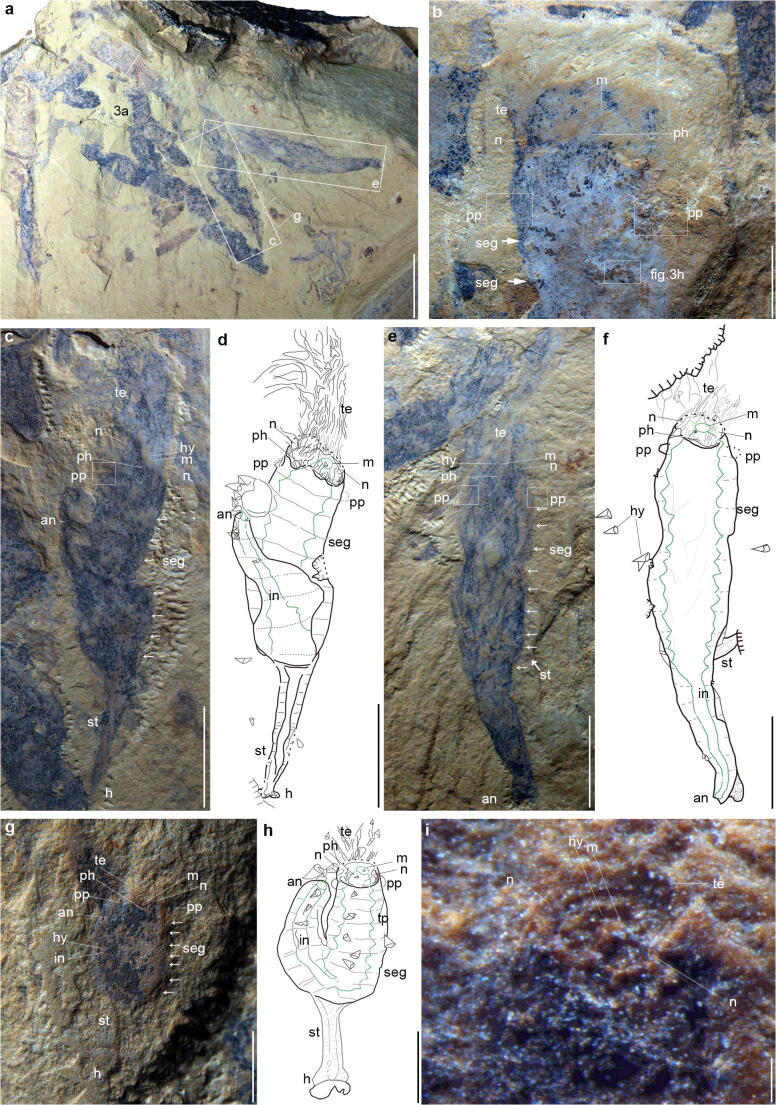
*Herpetogaster haiyanensis* n. sp. from the Chengjiang biota (Cambrian, series 2, stage 3) of China. **a** More than eight individuals preserved on a single slab. **b** A partial adult (YKLP 14407) preserved in dorso-ventral view, showing tentacles and pharyngeal pores (indicated by pp) at the anterior part of the body. **c** The holotype (YKLP 14404) preserved in lateral view. Segments are indicated by solid lines and demarcated by arrows. **d** Interpretive drawing illustrating the preserved structures in **c**. **e** A nearly complete, elongated adult (YKLP 14405) preserved in dorso-ventral view. Segments are indicated by dashed lines and demarcated by arrows. **f** Interpretive drawing illustrating the preserved structures in **e**. **g** A complete, coiled specimen (YKLP 14406), interpreted as a juvenile, preserved in lateral view. Segments are indicated by dashed lines and demarcated by arrows. **h** Interpretive drawing illustrating the preserved structures in **g**. **i** Close-up of the top of head segment in **g**; white solid lines indicate the notch, tentacle, and mouth, respectively. Te, tentacle; ph, pharynx; pp, pharyngeal pores; an, anus; in, intestine; seg, segment; st, stolon; h, holdfast; hy, hyolith; m, mouth; n, notch; stom, stomach; tp, triangular projection. Scale bars are 1 cm in **a**; 2 mm in **b**; 5 mm in **c**–**f**; 1 mm in **g**, **h**; and 100 μm in **i**

*Herpetogaster haiyanensis* n. sp. (Fig. 2a–i)

*Etymology*. After “Haiyan”, where the specimens were collected

*Holotype*. YKLP 14404 (Fig. 2c, d)

*Paratypes*. YKLP 14405-YKLP 14411

### Occurrence

Chiungchussu Formation, Yu’anshan Member, lower Cambrian (series 2, stage 3), *Eoredlichia*-*Wutingaspis* biozone, Haiyan section near Kunming, Yunnan Province, South China.

### Diagnosis

Species of *Herpetogaster* with bilateral symmetry, unevenly segmented body, ranging from curved to discoidal, coiled dextrally. Slender tentacles emerging from anterior most segment. Tentacles branching at the base and throughout the length of the tentacle, preserving over 100 branches. Trunk divided into two sub-sections, tapering towards the posterior end of the body, with at least 11 segments. Ventral and contractile adhesive stolon, with a terminal holdfast.

### Description

Most specimens (interpreted as adults) range in size from 21 to 24 mm, but a single specimen (interpreted as a possible juvenile) (Fig. 2g) is 3.8-mm long.

The specimens are laterally compressed and composed of a sub-cylindrical body separated into a head, with tentacles emerging from it, and a trunk that comprises about three-quarters of the total body length. A stolon with a darker inner and a lighter outer layer extends from the posterior part of the trunk.

**Fig. 3:**
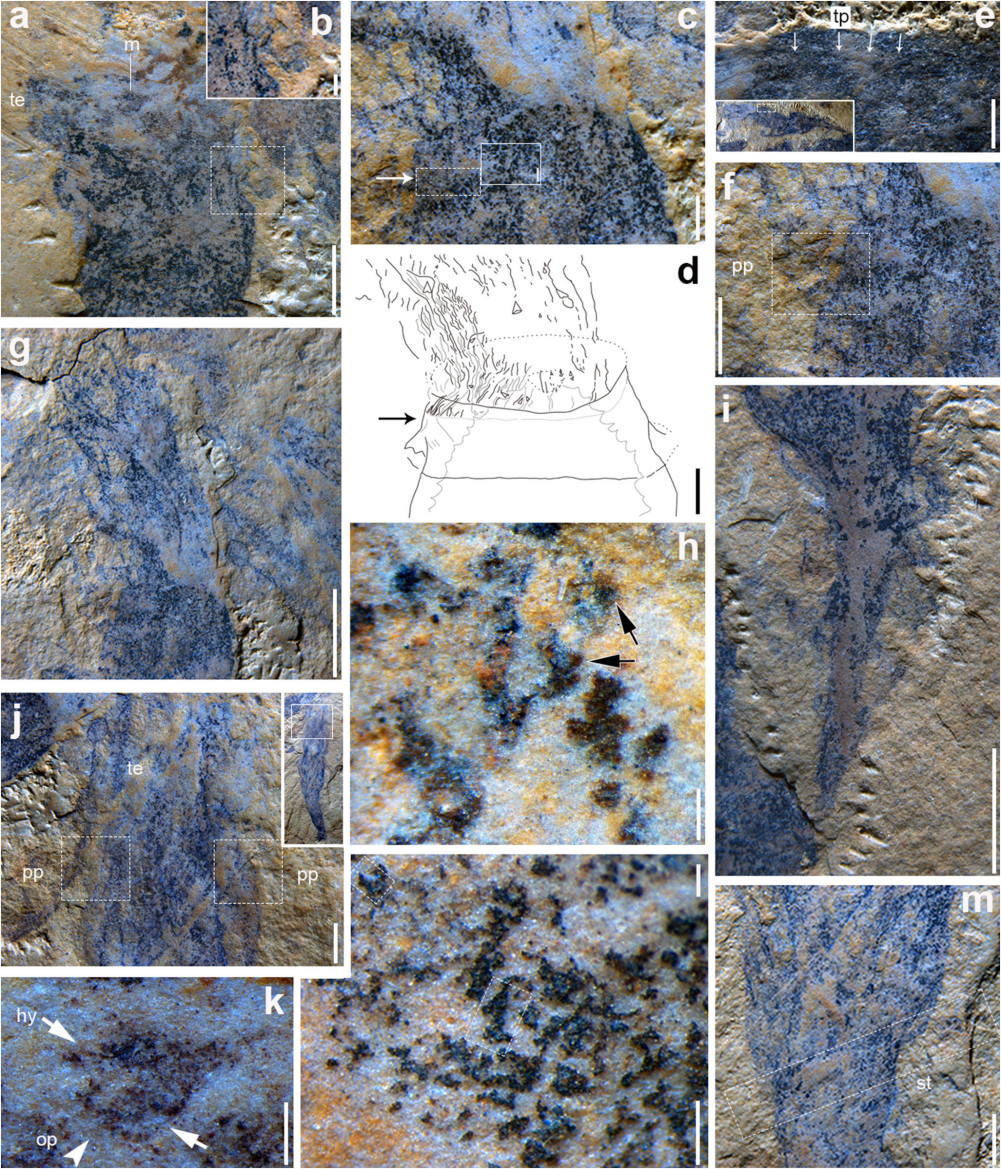
*Herpetogaster haiyanensis* n. sp. from the Chengjiang biota (Cambrian, series 2, stage 3) of China. **a** Close-up of the mouth, tentacles, and right pharyngeal pores (indicated by white dotted line) (YKLP 14408). **b** Close-up of the right pharyngeal pores from **a**. **c** Close-up of the mouth and tentacles in the holotype (YKLP 14404). **d** Interpretive drawing illustrating the preserved structures in **c**; the arrow indicates the base of tentacle crown. **e** Close-up of triangular projections (indicated by arrows) in the holotype (YKLP 14404). **f** Close-up of the left pharyngeal pore in the holotype (YKLP 14404). **g** Close-up of the tentacles in the holotype (YKLP 14404). **h** Close-up of the gut contents (mostly represented by black particles) of the paratype YKLP 14407. Two structures showing slight three-dimensional relief are interpreted as hyoliths (indicated by arrows). **i** Close-up of the complete stolon showing the darker preserved outer layer and the lighter preserved inner layer (YKLP 14405). **j** Close-up of the anterior of the paratype YKLP 14405, showing well-preserved tentacles and the pharyngeal pores. **k** Close-up of the mouth of the paratype YKLP 14408 preserving the oral peristome and a couple of hyoliths without operculum. **l** Close-up of the area just under the oral opening in the holotype (YKLP 14404) indicated in **c**. White frame shows a putative hyolith without operculum. **m** The posterior of the trunk of the paratype YKLP 14405, preserving a dislodged stolon (indicated by the white frame). hy, hyolith; op, oral peristome; pp, pharyngeal pores; tp, triangular projection. Scale bars are 1 mm in **a**, **e**, **f**, **j**, and **m**; 2 mm in **g** and **i**; 100 μm in **h** and **l**; 200 μm in **k**; 250 μm in **b**; and 500 μm in **c** and **d**

The holotype (Fig. 2c) is complete and 21-mm long in dorso-ventral view, including the tentacles and stolon, with the tentacles representing one-third (7 mm) of the length of the specimen. Another mostly complete specimen (Fig. 2e) preserved in dorso-ventral view is nearly straight, 24-mm long including the tentacles, and the stolon is mostly covered by the compressed posterior trunk (Figs. 2e, f and 3m).

In lateral view, the head preserves a pair of notches, which appear to represent the base of the tentacle crown (as indicated by a solid line and the letter *n* in Fig. 2d–i). The tentacles bifurcate with more readily visible branches at their base, around the anterior most head segment (Fig. 3c–j). There are about 5 branches per 100 μm in the holotype (Figs. 2c, 3c).

The trunk of the new species preserves a series of transverse bands that are interpreted as body segments, although the segmental boundaries in *H. haiyanensis* are not especially prominent. There are at least eleven segments, about 1.7-mm wide, in adult specimens (Fig. 2c). The lines are preserved on the dorsal and ventral sides (Fig. 2c, g), suggesting 360° annulation.

The digestive tract consists of the anterior mouth, pharynx, expanded stomach, and narrow intestine connecting with a terminal anus. In addition, a set of triangular projections (TP) suspended by mesenterial elements (Figs. 2c–g, 3d) are present. These are similar to the ones previously recognized in *H. collinsi* and *Eldonia ludwigi* and are repeatedly arranged along both inner walls of the visceral cavity. The mouth is located at the center of the anterior end of the head segment. In the holotype (Fig. 2c) the mouth is sub-circular, with a diameter of about 400 μm, and a hyolithid shell (about 250-μm long) is present (Fig. 2c). Immediately following, the mouth is a wider truncated cone-shaped structure interpreted as the pharynx that connects directly to a large stomach; the posterior part of the pharynx is about as wide as the stomach. In the holotype, the pharynx is about 1.6-mm wide at the posterior end, almost equal to the dimensions of the stomach (Fig. 2c). In the juvenile specimen (Fig. 2g, h), the diameter of the mouth is 100 μm. The oral peristome is visible in YKLP 14408 (Fig. 3k). Two structures (semicircular in lateral view) are symmetrically placed along the outer edges of the head segment; their diameter is about 300 μm in the holotype (Figs. 2c, 3f). Similar structures have tentatively been interpreted as pharyngeal pores (PP) in *H. collinsi* (Caron et al. 2010), and this interpretation appears to be confirmed by the new species (Figs. 2b, 3a–j). The PP are extremely fragile, and most specimens only preserve their imprints, rather than the actual pores. The diameter of the PP in YKLP 14405 is about 0.5 mm (Fig. 2e) and 0.6 mm and 0.4 mm in YKLP 14407 (Fig. 2b) and YKLP 14408 (Fig. 3a, b), respectively. In the juvenile specimen (Fig. 2g), they have a diameter of 120 μm. The stomach connects to the intestine around the tenth segment (Fig. 2c–g). The intestine terminates at the anus in the last segment (Fig. 2c, g). Some possible biological fragments can be observed near the anus (Fig. 2c–h). The stolon of *H. haiyanensis* extends outward from the tenth segment and consists of an inner and outer layer (Figs. 2c–g, 3i), which might be due to preservation conditions, as the separation is not visible in all stolons. The stolon of the holotype is 5-mm long (Fig. 2c). In the juvenile specimen, it is 1.2-mm long (Fig. 2g). A holdfast of indeterminate shape is preserved (Fig. 2c, g), and in the juvenile, it may be attached to an unidentifiable biological fragment (Fig. 2g, h).

The sub-cylindrical body can vary from coiled to straightened, and this variability may be attributable to taphonomic factors. In the relatively straight specimens (specimens that are dorso-ventrally compressed), the stolon is usually partially covered (Figs. 2a–f, 3m). When it is not visible, it may be covered, retracted, or broken off. By contrast, specimens that are curved possess well-preserved stolons, e.g. the holotype (Fig. 2c). In the holotype, the trunk bends outward from the end of the coelomic sac and appears coiled dextrally (Fig. 2c, d); a similar arrangement can be seen in the juvenile specimen (Fig. 2g, h).

### Remarks

The specimens are assigned to *Herpetogaster* based on the presence of anterior slender tentacles that are symmetrically arranged, the segmented body that can be either coiled or elongated, the pharyngeal pores, the digestive tract, stolon, and the terminal holdfast. Both *H. haiyanensis* and *H. collinsi* consist of a coiled dextrally segmented body. *Herpetogaster haiyanensis* preserves at least eleven segments and *H. collinsi* at least thirteen segments. Both species have a short head, although the head is slightly wider in relation to the body in *H. haiyanensis* (Figs. 2b, 3a–j). Several specimens of *H. haiyanensis* have straightened bodies, suggesting they may possibly have been more flexibile than *H. collinsi*, in which this preservation has not been documented*. Herpetogaster haiyanensis* bears tentacles with a greater number of branches (over one hundred branches present in the holotype) than those of *H. collinsi* (Caron et al. 2010; Kimmig et al. 2019b), suggesting that there might be differences in the feeding ecology of the two species. The stolon is in comparable positions in both species but appears to be separated into an outer and inner layer in *H. haiyanensis*, instead of a single layer as in *H. collinsi* (Caron et al. 2010; Kimmig et al. 2019b). A stolon separated into two layers corresponds to the condition in *Siphusauctum* (see O’Brien and Caron 2012; Kimmig et al. 2017), each has a holdfast and in each the digestive tract consists of an anterior mouth, pharynx, and large stomach that ends in an anus in the terminal segment. But it might be that the two layers of the stolon in *H. haiyanensis* might be a taphonomic relict.

**Fig. 4:**
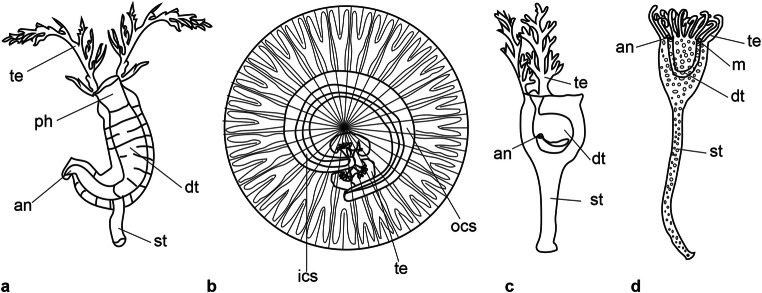
Line drawings of four possible relatives of *Herpetogaster haiyanensis* n. sp. **a** *Herpetogaster collinsi* (modified from Caron et al. 2010). **b** *Eldonia ludwigi* (modified from MacGabhann 2012). **c** *Phlogites longus* (modified from Hou et al. 2006). **d** *Cotyledion tylodes* (modified from Zhang et al. 2013). an, anus; dt, digestive tract; ics, inner coiled sac; ocs, outer coiled sac,; m, mouth; ph, pharynx; st, stolon; te, tentacles

Caron et al. (2010) and Kimmig et al. (2019b) provided comparisons between *Herpetogaster* and several taxa, including *Eldonia* and *Phlogites*, such that a detailed discussion need not be repeated here. However, key differences between *Eldonia* and *Herpetogaster* include the former possessing a more coiled digestive tract and the latter having a stem that attaches it to the seafloor. Key differences between *Phlogites* and *Herpetogaster* include the former having broader, less numerous tentacles, and a thicker stolon. Relative to *Cotyledion tylodes*, Luo and Hu in Luo et al. (1999), *Herpetogaster* differs in the arrangement of tentacles (in the former they comprise a small frill around the anterior margin), in the absence of the round sclerites covering the body in the latter, and the absence of a stolon in the former (Fig. 4).

Numerous specimens on a single slab suggest that *H. haiyanensis* may have had a gregarious lifestyle, and differences in the length and extension of the stolon suggest that this structure might have been retractable. The excellent preservation, including a stolon in most specimens, suggests little to no transport and rapid burial. Similar to *H. collinsi*, small hyoliths are preserved in association (Figs. 2c–h, 3h–l). In addition, hyoliths and unidentifiable animal pieces can be found within tentacle clusters and the gut of some specimens (Fig. 3h, l). Hyolith conches are found in the slender tentacle cluster, in the mouth, and in the digestive tract. While it is possible that the animal actually fed on hyoliths, it is although possible that the hyoliths themselves aggregated around the specimens (R. Bicknell, pers. comm. 2020) or that they accumulated postmortem through currents, as no opercula or helens can be identified. If *H. haiyanensis* fed on the hyoliths or they aggregated themselves around the specimens, it could provide support to Kimmig and Pratt’s (2018) and Sun et al.’s (2018) suggestion that hyoliths were mobile.

## Conclusions

New fossil discoveries from Cambrian BST deposits continue to increase our understanding of the range of animal life extant during and shortly after the Cambrian radiation. Studies on fossils from these deposits have critically enhanced understanding of ecdysozoan diversity and relationships, and a clearer picture is beginning to emerge for Deuterostomia as well. For instance, the documented diversity of early deuterostomes in Cambrian BST deposits continues to climb. This study also suggests that there may be a close biogeographic relationship between *Herpetogaster* from the Cambrian stage 3 of South China and the Cambrian stage 4 and Wuliuan stage of Laurentia (for a recent discussion of biogeography of Cambrian BST deposits in South China and Laurentia see Pates et al. 2019). However, whether this biogeographic relationship is related to patterns of faunal migration or instead simply reflects an incomplete knowledge of *Herpetogaster* diversity and distribution awaits future discoveries.
